# P08-06 MOVEluencer: a project to promote physical activity during the COVID-19 pandemic in a rural community setting

**DOI:** 10.1093/eurpub/ckac095.119

**Published:** 2022-08-29

**Authors:** Peter Holler, Silvia Tuttner, Frank Michael Amort

**Affiliations:** Institute of Health and Tourism Management, FH JOANNEUM, Bad Gleichenberg, Austria

**Keywords:** physical activity, promotion, COVID-19 pandemic, rural community, active mobility

## Abstract

**Background:**

An appropriate level of Physical Activity (PA) is important for both physical and mental health and has also been suggested as a protective element against COVID-19. However, even before the outbreak of the COVID-19 pandemic, there were already a high number of physically inactive people in Austria. Due to ongoing pandemic-related social restrictions, not only a further increase of inactivity is suspected, but also the promotion of PA is currently a major challenge. Therefore, the project “MOVEluencer” which has been running since July 2021, follows a multidimensional PA promotion approach that is less affected by the presence of any social restrictions.

**Methods:**

The project was realized in six rural communities in Styria (a province of Austria), which were selected based on inequality factors related to health and PA. The target groups are physically inactive residents, particularly families and elderly. Overall, the project measures are based on a participatory approach and are implemented in three sections (phase 1 -3).

**Results:**

Phase 1 (July - December 2021) represented an analysis phase in which a photo-challenge was conducted. The aim of the challenge was to identify resources and opportunities for improving active mobility and PA in the communities based on photos taken by residents. Sixty-seven people (43% female) participated in the challenge and submitted a total of 268 photos which were disseminated via the project-specific social media channels. Moreover, in phase 1 working groups represented by members from the project target groups were established. Phase 2 is currently being implemented (since January 2022). One measure is to recruit and train walking buddies to provide regular walks and simple strength exercises along the walking-way. A further measure represents outdoor-educational events, following an interactive learning approach to improve the populations awareness of active mobility and PA in general. During these events, simple tests that assess components of health-related fitness are also offered. Phase 3 will implement target group-specific PA programs currently being developed in the working groups.

**Conclusion:**

Promotion of PA remains significant, especially during a pandemic and in rural communities, as awareness of the benefits of PA in this setting is still low.

